# HusMorph: a simple machine learning app for automated morphometric landmarking

**DOI:** 10.1093/conphys/coaf073

**Published:** 2025-10-22

**Authors:** Henning H Kristiansen, Moa Metz, Lorena Silva-Garay, Fredrik Jutfelt, Robine H J Leeuwis

**Affiliations:** Department of Biology, Norwegian University of Science and Technology, Høgskoleringen 5, 7034 Trondheim, Norway; Department of Computer Science, Norwegian University of Science and Technology, Høgskoleringen 5, 7034 Trondheim, Norway; Department of Biology, Norwegian University of Science and Technology, Høgskoleringen 5, 7034 Trondheim, Norway; Department of Biology, Norwegian University of Science and Technology, Høgskoleringen 5, 7034 Trondheim, Norway; Department of Biology, Norwegian University of Science and Technology, Høgskoleringen 5, 7034 Trondheim, Norway; Department of Biological and Environmental Sciences, University of Gothenburg, Medicinaregatan 7B, 41390 Göteborg, Sweden; Department of Biology, Norwegian University of Science and Technology, Høgskoleringen 5, 7034 Trondheim, Norway

**Keywords:** Artificial intelligence, automation, images, machine learning, morphometrics, phenotyping, user-friendly

## Abstract

Manually obtaining the length and other morphometric features of an animal can be time-consuming, and consistent measurements are challenging with large datasets. By leveraging high-throughput computing power and machine learning–based computer vision, such phenotypic data can be rapidly collected with high accuracy. Here we present HusMorph, a novel application with a simple and intuitive graphical user interface (GUI), based on the same machine learning method used in other pipelines such as ML-morph. It consists of an all-in-one package with the goal of making machine learning easy to use for non-experts. The user starts by setting any number of landmarks on a set of photos captured with a standardized setup. From this set, a machine learning model is generated by automatically and randomly searching for the best performing parameters. Next, the user can apply the model to predict landmarks on new standardized photos and visually confirm and export the results of the predictions. For measuring length between landmarks, an additional feature allows for detecting a scale bar for each photo to convert the length from pixels to a metric unit. Our application has been validated and applied to extract standard length from 1935 photos of zebrafish and performs with ~99.5% accuracy compared to manual measurements.

## Introduction

Morphometric measurements are essential in most disciplines of biology, and important for advancing our understanding of evolution, species identification and taxonomy, genotype–phenotype relationships, effects of various factors and environmental stressors on body condition, biomechanics and palaeontology (Rohlf and Marcus, 1993; Klingenberg, 2010; Rowiński *et al*., 2015). In recent years, there is a growing trend of large-scale morphometric datasets being collected by biological researchers, such as repositories consisting of thousands of images (van der Linde *et al*., 2018; Edlund *et al*., 2021). Traditionally, however, morphometric information is still extracted through manual annotation, which is time-consuming and labour-intensive. Furthermore, inter- and intra-observer measurement errors in landmark placements can lead to inconsistencies and faulty estimates (von Cramon-Taubadel *et al*., 2007).

Recent advances in artificial intelligence (AI) have opened up vast possibilities in the field of biology, including the potential to increase the scale, complexity, accuracy, efficiency and reproducibility of phenotypic data collection (Wang *et al*., 2023). Computer vision is a prominent AI-based technology that allows for automated landmark placement and morphometric data collection, reducing variability introduced by manual annotation and improving the consistency of measurements across large datasets and significantly decreasing processing time. Machine learning (ML) algorithms and methods common in computer science facilitate quick and accurate morphometric landmarking for complex dataset analyses that were previously impractical (He *et al*., 2024). In fact, computer vision may become a fundamental part of the toolkit for biologists interested in extracting phenotypic data from images (Lürig *et al*., 2021).

Given that machine learning–based phenotyping pipelines and infrastructure available to date are implemented in Python (e.g. Porto and Voje, 2020), a sufficient level of Python coding and machine learning knowledge is required for biologists to use these resources. To remove this barrier and increase the accessibility of machine learning–based landmarking of photos to non-experts, a graphical user interface (GUI) can be an important attribute of such a research tool. Additionally, open-source software has the benefit of being available to all researchers regardless of financial resources. Here, we present HusMorph, a free stand-alone and open-source machine learning application with a graphical user interface, automation of functions, and other user-friendly features to extract morphometric measurements from images. This application runs as a stand-alone executable on both Windows and Mac computers without requiring Python environment management. This makes it unique from comparable tools as it enables anyone in the biological field to automate high-throughput morphometric analyses.

The built-in machine learning procedure of HusMorph is well established in the computer vision field. More advanced variants to this approach also exist—e.g. those that combine object detection with landmark prediction (Porto and Voje, 2020) or 3D-pose estimation with tracking (Mathis *et al*., 2018). However, in our view, users are still required to have prior expertise in coding or machine learning to successfully build and use such machine learning pipelines. We specifically designed HusMorph to be accessible to users without specialized technical knowledge, by automating the parameter optimization of the machine learning model and packing the method in a user-friendly application with an intuitive graphical user interface. This application is not intended to replace experts in the machine learning field; rather, it serves as a supplementary tool, enabling high-throughput analysis without the need for specialized technical knowledge. While expert users can also benefit from its capabilities, its primary focus is to support those without advanced expertise in the field. By integrating complex machine learning processes into a program with an easy to use graphical user interface, HusMorph facilitates automation in phenotyping, allowing researchers in the morphometric community to quickly assess biological structures in images at a large scale regardless of technical background.

## Materials and Methods

We developed HusMorph as an accessible application with a user-friendly graphical user interface based on Python code. This interface allows users to quickly place and view landmarks on images with minimal user input (i.e. number of button presses). It also enables automated landmark placements using computer vision and machine learning techniques, primarily leveraging OpenCV 4.10.0.84, dlib 19.24.6, Optuna 4.1.0 and matplotlib 3.9.3 libraries. Dlib is the machine learning library used, and the application uses its standard central processing unit (CPU) setup. The interface is designed with intuitive and interactive elements (e.g. buttons and drop-down menus). Furthermore, the program is packaged as an executable file, allowing users to run it directly without needing to set up a Python environment or install additional dependencies, making it easy to use without requiring any coding knowledge. The application can be downloaded free from charge through GitHub (https://github.com/HenHus/Husmorph), and specific user instructions are available on the same web page. In the following sections, we provide a detailed explanation of the key features of HusMorph (Table 1), and evaluate its performance on different datasets to validate the method and demonstrate its reliability. We tested the software on photos of fishes, although it should perform equally well on many other organisms or biological structures.

**Table 1:** Recommended dataset specifications and built-in machine learning procedure steps and features used for automated landmarking with the HusMorph application

**Component**	**Description**
**Input image recommendations**	
Quality	Standardized rotation, flipping and scaling
Number	≥100
Resolution	≤2 megapixels
Background	Distinct from object, ideally homogeneous
Other	Consistent lighting and focus
**Built-in machine learning procedure steps and features**	
Manual landmarking in the training set	Ground truth in the training dataset is established through expert manual landmark placements.
Machine learning model training and testing	Machine learning models are generated from the set of images with the manually placed landmarks.
Automated landmarking and visual confirmation	The model automatically places predicted landmarks on unseen images, which users can review to confirm accuracy.
Automated scale bar detection	Optional automated scale bar detection script converts pixels to length units for each image in batch mode.
Export of predicted landmarking results	Predicted landmark results can be conveniently exported in CSV format.

**Figure 1:**
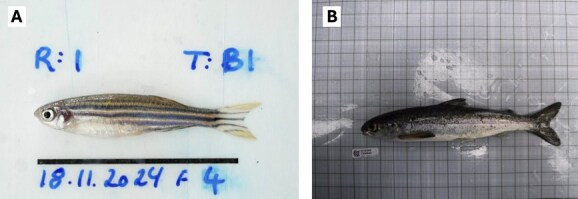
Examples of fish images suitable for machine learning training. (A) Zebrafish (*Danio rerio*) against a white background with few other image elements. The scale bar is for length calibration. (B) Atlantic salmon (*Salmo salar*) against a slightly more complex background with light reflection, but with sufficient contrast from the organism of interest to allow for automation of landmarking.

### Image requirements

Machine learning training is easier if the images are optimized for the process (Table 1). A homogenous background with a colour that is distinct from that of the organism is advantageous. We have mainly tested the process using zebrafish (*Danio rerio*) placed on their ventral side against a solid white background (Fig. 1A). However, machine learning can also work well with images containing slightly more complex backgrounds (Fig. 1B). Additionally, the method employed in the training process is sensitive to rotation, flipping, and scaling, indicating that better image standardization leads to improved results.

As the machine learning training is computationally intensive, very high-resolution images require too much hardware without necessarily benefiting accuracy in landmark placements. Therefore, we recommend downscaling the images to match the processing power of the computer hardware available prior to training. We have used images ~1000–2000 pixels on the long side and recommend this as the highest resolution to be used for current processing power. This image resolution is amenable to training large sets (i.e. hundreds) of images on regular desktop computers and modern laptops. Very fast stationary computers (e.g. server clusters, workstations, gaming computers) may handle training sets with higher resolution images.

The number of images needed for successful training depends on the complexity and variability of the dataset, as well as the desired accuracy. Generally, a larger number of photos improves the accuracy of automated landmarking by increasing the diversity of training samples. To determine the optimal balance, we tested different training set sizes to identify the point where accuracy becomes sufficient and additional training data yield minimal improvement.

### Computer hardware requirements

Training of machine learning algorithms is a computationally demanding process that requires both high-performance hardware and/or extended training duration. The accuracy of landmark predictions is highly dependent on the size of training datasets, with larger dataset and longer training times, generally leading to better results. To ensure efficient training, we primarily used a desktop computer with a high-performance processor (Apple Mac Studio M2 Ultra). Additionally, we tested the training on various MacBook and Windows laptops. On modern laptops, a session of machine learning training can take ~1–2 days of continuous training, depending on factors such as dataset size, image resolution, number of machine learning iterations, and hardware specifications. It is important to note that dedicating the whole processor to training may cause the computer to become hot and slow to perform other processes. However, machine learning training is a one-time process. Once the machine learning training is complete, it becomes substantially less computationally demanding, allowing it to place landmarks on large datasets of new images within seconds, even on standard laptops.

### Automation of the machine learning process

To eliminate the need for users to possess an in-depth understanding of machine learning, the training parameters that affect the outcome are not manually adjusted. Instead, nine parameters are automatically optimized using the Optuna library—an open-source hyperparameter optimization framework that employs advanced algorithms to efficiently search for optimal parameter configurations (Akiba *et al*., 2019). Based on the training and testing conducted in this study, we have established a range for each parameter that performed well. Within these defined ranges, Optuna autonomously explores the optimal combination, avoiding the exhaustive search required by traditional methods like grid search—especially when dealing with rational-valued parameters. This streamlined approach reduces computational time and enhances overall efficiency.

Once the training process is initialized, an Optuna ‘study’ object is created within the memory that temporarily stores a collection of ‘trial’ objects: a representation of a single execution of the train_shape_predictor() function from the dlib library, along with the hyperparameters used and the performance score. The application automatically splits the dataset and performs a 5-fold cross validation for each trial, before the mean performance from the cross validation returns back to the study object and gets saved. Since the shape predictor returns with the unit pixel deviation, the Optuna study adaptively searches for future hyperparameter combinations for the shape predictor trainer to lower the return value. The only parameter set by users are the numbers of trials to include in a study, allowing them to balance the likelihood of finding a high-performing trial and minimizing the total time required to achieve the desired accuracy. Finally, one model is derived from the best performing trial with the lowest mean fold deviation, ensuring optimal accuracy.

### Setting and viewing landmarks

One of the key features of the HusMorph app is the function that allows users to efficiently place landmarks manually on images, providing a workflow similar to commonly used tools like ImageJ. It simplifies batch processing, as it saves landmarks in an XML format for seamless downstream use. While it can act as a stand-alone feature for quickly placing landmarks on smaller batches of images, its primary role is for creating the training dataset for machine learning (Table 1).

Landmarks are represented as coordinate points on the image, with the origin located in the upper-left corner. The coordinate data are saved directly in an XML format, ensuring compatibility with machine learning workflows. Users can revisit and view the landmarks at any time, as the saved XML file contains all the necessary information for displaying the landmarks. This feature is particularly helpful for visually assessing the performance of machine learning models. By displaying the landmarks on the images, it provides a clear, intuitive way to evaluate accuracy—something that can be challenging to grasp from numeric measures like pixel deviation or percentage values alone.

**Figure 2:**
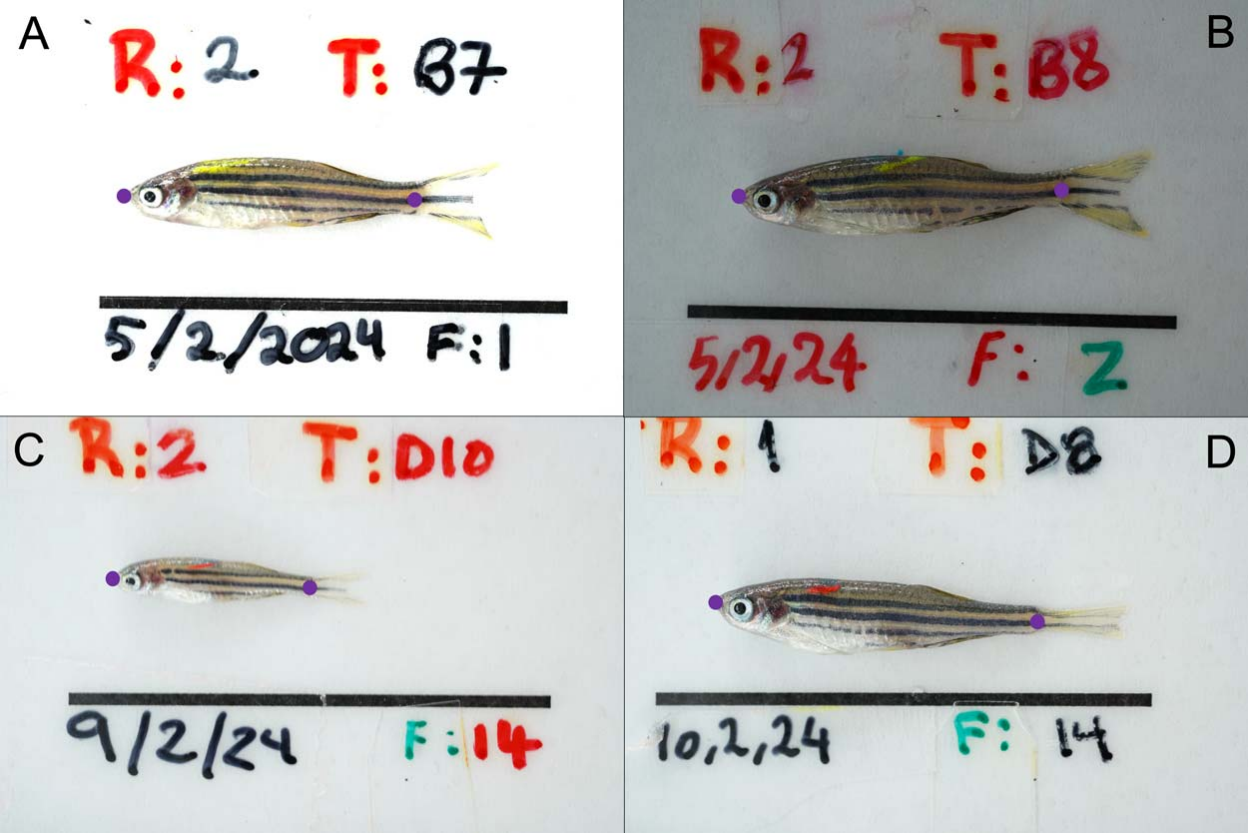
A selection of zebrafish images illustrating the variability within the landmark dataset used to evaluate app performance. (A) Slightly overexposed image, (B) highly underexposed image, (C) slightly blurred image featuring a very small zebrafish, and (D) zebrafish with an irregular, unspread caudal fin. The landmarks at the tip of the snout and the hypural joint of the caudal peduncle were used for determining standard length. The scale bar below the zebrafish facilitates the conversion of pixel measurements to millimetres.

### Landmark accuracy

To quantify the accuracy in landmark placements, we measured the Euclidean distance in pixels between each landmark’s true location and that predicted by the model, which is then normalized against the diagonal pixel distance of the total image. The percentage error of the diagonal reflects how well the model handles the input image itself. This is a different measure for accuracy compared to the deviation relative to the total length of the biological structure. Normalizing by the length of the structure is also common, although it fails to take into consideration the extent that the structure fills up the image frame. Therefore, by setting deviations as a percentage of the image diagonal—the biggest error possible—allow for an unbiased comparison of the prediction errors across different datasets and species.

To further validate the results, we will compare the model’s performance with intra-observer error. Intra-observer error quantifies the variability in landmark placement when the same human expert annotates the same images on different occasions, serving as a baseline for manual accuracy. By comparing the model’s deviation metrics to this error, we can assess whether the automated predictions achieve a level of consistency comparable to that of an expert. This evaluation tests if the model’s performance falls within an acceptable range of accuracy, examining its robustness and practical applicability for high-throughput morphometric analyses.

### Scale bar detection

The HusMorph machine learning application includes a convenient extension for automatically detecting and measuring scale bars in images (Table 1). This feature provides an efficient way to convert pixel distances into more meaningful units such as millimetres. Images first undergo automatic pre-processing to enhance line features, after which the Hough Line Transform is applied to detect lines based on user-defined parameters for threshold, minimum line length, and maximum line gap. With minor coding adjustments tailored to the specific scale bar, the tool can be customized to ensure accurate calibration across all images. This functionality is valuable because even small movements of the camera or subject positions can cause variations in scale from image to image.

### Testing dataset size and accuracy on a standardized dataset

To evaluate the performance of the application, we conducted tests using image datasets of varying sizes: 50, 100, 250 and 500 images. All images were resized to 1800 × 1200 pixels to analyse the relationship between dataset size and prediction accuracy. The images were randomly selected from a pool of 1900 standardized images of zebrafish. Each image featured labelled zebrafish on a white background, all oriented in the same direction and positioned relatively horizontally with an extended caudal fin (Fig. 2). The dataset was collected as a part of a large-scale experiment that we will refer to as the selection experiment; a multigenerational experiment with thousands of images for each generation. Zebrafish were anaesthetized in buffered MS-222 (300 mg/l) prior to being photographed. Two camera set-ups were used, each with an LED tent (LST40, Godox, China) to control lighting conditions and with the camera (ILCE-7C, Sony, Japan) and lens (SEL90M28G, Sony, Japan) mounted on a table with one of two stands (DT-30, SmallRig, China or 2089, Ulanzi, China).

The dataset introduced variability, including being gathered by multiple people, two different cameras, differences in zebrafish size (ranging from 10 to 28 mm in standard length), variations in image clarity (e.g. focus and blur) and changes in lighting and exposure. This variability enabled evaluating the application robustness under varying image qualities and conditions in a standardized dataset.

Each image included two landmarks: one at the tip of the snout and another at the point where the caudal fin begins. Figure 2 illustrates these placements (blue-filled circles). The two landmarks entailed different levels of detection difficulty. The snout landmark (LM1), located at the edge of the background, was easier to identify, while the caudal peduncle landmark (LM2), positioned at the hypural joint, was more challenging to detect.

### Testing a field dataset

**Figure 3:**
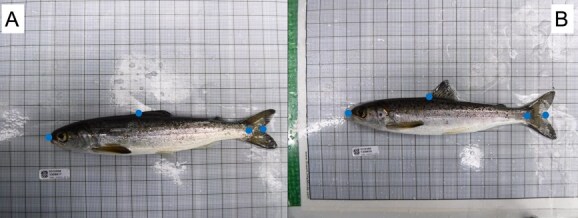
Sub-sample of a field dataset of Atlantic salmon images used to evaluate app performance. Each image (A and B) shows four dots, representing the four landmarks used; at tip of the snout, at the hypural joint of the caudal peduncle, at the fork of the tail fin and at the base of the dorsal fin.

To further assess the robustness of the landmark prediction, we conducted tests using field images of Atlantic salmon parr and smolts (Moccetti *et al*., 2025), which had minimal standardization (Fig. 1B). Salmon were anaesthetized and imaged with a compact camera (FinePix XP130, Fujifilm, Japan) that was handheld at ~30 cm directly above the fish. A total of 261 images with a resolution of 1200 × 900 pixels were analysed, with landmarks placed at the tip of the snout, the base of the caudal fin, the fork of the tail and the base of the dorsal fin (Fig. 3). After completing the training process, an additional set of 29 previously unseen images was used for final validation to further evaluate the model’s performance.

### Ethical declarations

The husbandry and experimental procedures were conducted in line with the Norwegian Welfare Act and followed the Norwegian Food Safety Authority’s safety protocol (permit number 8578).

## Results

### Image dataset size and accuracy on a standardized dataset

The accuracy was measured by measuring the machine learning performance on 1548 unseen images of zebrafish, and the accuracy was compared with intra-observer error where the same person manually placed landmarks on the same dataset 9 months apart.

**Figure 4:**
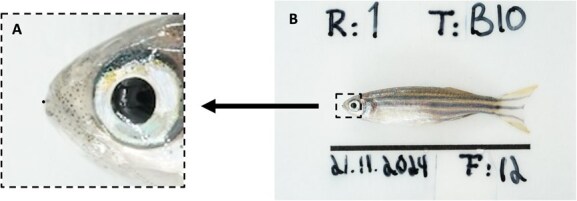
Visual representation of the accuracy of automated HusMorph landmarks placed on zebrafish images. Image A is an enlargement of the full image shown in Image B. The black circle in Image A illustrates a 5-pixel deviation—a level of accuracy achieved after training on 500 images across 200 trials.

**Figure 5:**
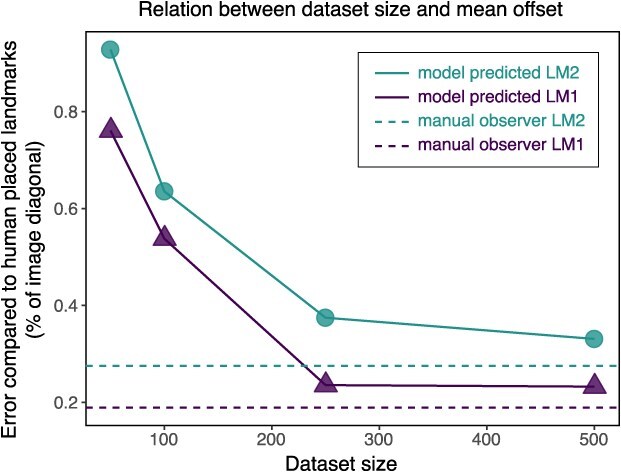
Comparison of the placement error between intra-observer error on the zebrafish dataset, marked as ‘manual observer’, and automated machine learning landmark placements, marked as ‘model predicted’, as a function of dataset size. Two types of landmarks are tested, the simplest landmark (LM1) (triangles) shows high accuracy for both human and machine learning landmarking, while the more difficult landmarks (LM2) (circles) are overall more challenging to place.

All models performed with accuracy <1% of image diagonal on the unseen images (Fig. 4). The results showed that the dataset of 50 images has the highest mean error both for LM1 and LM2 (0.76 and 0.92% of image diagonal respectively, or 16 and 20 pixels), and drastically dropped to dataset size of 100 (0.54 and 0.64% of image diagonal, or 12 and 13 pixels), before it dropped further for size 250 (0.24 and 0.37% of image diagonal, or 5 and 8 pixels), and stabilized towards the dataset of size 500 (0.23 and 0.32% of image diagonal, or 5 and 7 pixels). LM1 had overall less error compared to LM2 (Fig. 5). When compared to intra-observer error—0.19% of image diagonal (4 pixels) for LM1 and 0.28% of image diagonal (6 pixels) for LM2—the model trained on 500 images was only 0.04% worse for both landmarks, approaching the theoretical maximum performance expected from a machine learning model.

**Figure 6:**
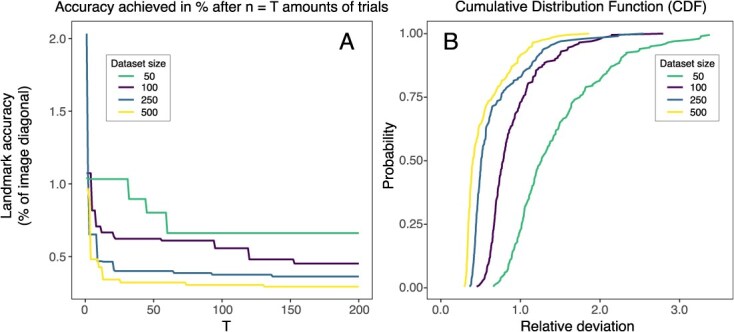
(A) Machine learning landmark placement accuracy is dependent on zebrafish image dataset size and the number of trials (*x*-axis). (B) Cumulative distribution function depicts the probability of reaching a certain deviation relative to the image diagonal using various image batch sizes. Image shows that larger training datasets have a higher chance of high accuracy. The accuracy reflects the results on the validation set.

Of the 200 trials conducted for each dataset, the dataset of size 50 exhibited significant improvements in the early trials but reached a performance plateau after trial 60. In contrast the dataset of size 100 demonstrated a more gradual improvement throughout the study. Notably, the datasets comprising 250 and 500 images outperformed the smaller datasets, achieving high accuracy within the first 25 trials, with only marginal gains thereafter (Fig. 6A).

Figure 6B illustrates the probability of achieving various levels of deviation. Steeper curves indicate a higher likelihood of obtaining a model with low deviation. The figure demonstrates that datasets of 250 and 500 images have a >80% chance of finding a model with <1% relative deviation. Sets of 100 images have ~70% chance whereas a dataset of 50 images has a substantially lower chance of reaching the same level of accuracy. Notably, the small difference in performance between the 250- and 500-image datasets suggests that a dataset of ~250 images may offer the best balance between computational effort and accuracy.

For the selection experiment, we were able to train a model using 1935 images of zebrafish, achieving an accuracy of 0.18% of the image diagonal by following the same procedure provided by the application. To further assess the model’s accuracy, a visual inspection of 6966 predicted landmarks across 3483 previously unseen images was conducted. The results indicated that 99.5% of the predicted landmarks were visually indistinguishable from those that would have been placed manually by an expert.

### Testing a field dataset

Based on the evolution of the study performed on the zebrafish dataset, we decided to make a study of 100 trials on the Atlantic salmon field dataset, since the performance seemed to stabilize after this point. On the 29 unseen images, the trained model achieved a mean error of 0.40% of image diagonal (6 pixels) per landmark. Intra-observer error was measured on the same 29 images with 1 day between the samplings, with a mean deviation of 0.21% of image diagonal (3 pixels).

## Discussion

Here, we show that our machine learning–based HusMorph application can automatically place landmarks with a high accuracy on par with human experts. Using training sets of 100–250 photos, the application achieves a sufficient level of accuracy (5–14 pixels of deviation) from the optimal landmark location on high-definition images for most biological purposes. A larger training dataset (more images) can further enhance accuracy (to ~6 pixels deviation), although it requires a higher computational effort (i.e. longer training time). We also show that bigger datasets have a very high chance of generating a well-performing model that achieves good accuracy. It is generally not worthwhile to apply machine learning to smaller datasets (<100 images), as the accuracy was poor with training on 50 images. For smaller projects we therefore recommend placing the landmarks manually. However, beyond a dataset size threshold of ~100 images HusMorph offers a powerful solution to produce high-quality landmarks and automate morphometric data collection.

As expected, we found that the machine learning models detected simple (high contrast) landmarks more easily than complex (low contrast) landmarks regardless of dataset size. This suggests that the difficulty of landmark recognition (e.g. contrast to background and complexity of the landmarks) are crucial factors determining error rates and the level of accuracy. We demonstrate this when testing HusMorph to detect both fork length and standard length in fishes. Both measures use the tip of the snout as the anterior landmark; however, fork length uses the anterior end of the middle caudal fin rays, whereas standard length uses the hypural joint of the caudal peduncle. There is usually a clear contrast between caudal fin and background (Fig. 1), while differences in coloration and structure between caudal peduncle and caudal fin are much more subtle. Consequently, standard length landmarks are more difficult features to predict with machine learning. We should note, though, that it is common for human observers to have a similar difficulty in placing standard length landmarks with high accuracy (Carlander and Smith, 1945).

A major advantage of automating morphometric data collection from images is the reduction of systematic biases and errors inherent in human observation (Marsh and Hanlon, 2007; Holman *et al*., 2015). For instance, human observers that have preconceived expectations of results may subconsciously skew landmark placements in favour of this outcome. Further, the location and accuracy of landmarks placed by observers may drift over time, especially when morphometricians spend many days to collect a dataset, which causes disparity between measurements made across a timespan. Finally, when multiple annotators are involved, there is a risk of increased data variance or inter-observer errors. Therefore, an automated approach is preferable in many contexts (Jutfelt *et al*., 2017; Clark *et al*., 2020; Clements *et al*., 2022). Our machine learning–based HusMorph application effectively achieves such automation, minimizes manual annotation errors and eliminates human biases.

The dlib library used in our machine learning software differs from typical convolutional neural network-based predictors using, e.g. Tensorflow or PyTorch, which typically train on large datasets containing thousands of input images and are harder to fine-tune (Xue *et al*., 2021). Predictive models developed that way are generally more robust, exhibiting higher tolerance to image transformations such as rotation, scaling and flipping, which can be weaknesses of our dlib library–based approach. Graphical processing unit (GPU)-accelerated training is not well supported in dlib either, which led us to not include this feature in our application. Nevertheless, we regard these trade-offs as favourable for most use cases, given the library’s simplicity, accessibility and the comparatively far smaller dataset requirements compared to alternative approaches. Most research studies in biology neither require nor have access to the resources that would support a very high level of image versatility. HusMorph is also more easily run on many types of computers thanks to CPU-based training. Therefore, for moderately sized (i.e. hundreds of images) and standardized datasets that are common in biological studies, the dlib library that HusMorph uses offers a great compromise between tolerance to variation in image quality and machine learning effort.

## Conclusions

The open-source HusMorph application allows researchers to quickly automate morphometric data collection from large sets of images. The program has an easy-to-use graphical interface and therefore allows anyone to employ the power of machine learning to their morphometric research questions, with a minimal learning curve. We show that the machine learning model can reach similar accuracy to human observers through training on a few hundred images over a day or two on a normal PC or Mac computer. We therefore hope that the HusMorph application will benefit researchers by increasing speed and ease of data collection, while at the same time promoting accuracy through reductions in biases and variation from human observation.

## Data Availability

The HusMorph application, user instructions, scripts and example image files can all be found on GitHub (https://github.com/HenHus/Husmorph).
